# IgM response against amyloid-beta in aging: a potential peripheral protective mechanism

**DOI:** 10.1186/s13195-018-0412-9

**Published:** 2018-08-16

**Authors:** Sudhanshu Agrawal, Edsel M. Abud, Shikha Snigdha, Anshu Agrawal

**Affiliations:** 1 Division of Basic and Clinical Immunology, Department of Medicine, University of California, Irvine, Irvine, CA 92697 USA; 2 UCI-MIND, University of California, Irvine, Irvine, CA 92697 USA

**Keywords:** Dendritic cells, Amyloid beta, Human, IgM antibody

## Abstract

**Background:**

The immune system plays a major role in the pathogenesis of age-related dementia, including Alzheimer’s disease (AD). An insight into age-associated changes in the immune response to amyloid-beta (Aβ) in individuals without AD may be beneficial in identifying mechanisms preventing accumulation of Aβ.

**Methods:**

We examined the response of human monocyte-derived dendritic cells (DCs), T cells, and peripheral blood mononuclear cells (PBMCs) from healthy aged and young subjects to Aβ peptide 1–42, Aβ fibrils, and recombinant, nonaggregated tau-4 protein with a view to understand the role of peripheral immunity in AD.

**Results:**

Our studies revealed that DCs from healthy aged subjects display weak reactivity towards the Aβ peptide and no reactivity towards Aβ fibrils and tau compared with their young counterparts. An analysis of old and young PBMCs revealed that there is no significant T-cell memory against Aβ peptide, fibrils, or tau. Remarkably, the plasma levels of IgM antibodies specific to Aβ peptide 1–42 were significantly increased in aged subjects compared with young subjects, while IgG levels were comparable. Aβ peptide-specific IgM and IgG levels were also determined in the plasma of AD subjects compared with age-matched controls to demonstrate that the immune response against Aβ is stronger in AD patients. A decline in Aβ peptide-specific IgM antibodies was observed in AD patients compared with age-matched controls. In contrast, the levels of IgG as well as interleukin-21, the major cytokine involved in class switching, were increased in AD and patients with mild cognitive impairment, indicating a strong immune response against Aβ.

**Conclusions:**

Collectively, low immunogenicity of Aβ in healthy controls may prevent inflammation while the generation of specific IgM antibodies may help in the clearance of Aβ in healthy subjects.

**Electronic supplementary material:**

The online version of this article (10.1186/s13195-018-0412-9) contains supplementary material, which is available to authorized users.

## Background

Aging is one of the most important risk factors for developing dementia, including Alzheimer’s disease (AD) [1, 2]. One of the hallmark pathologies of late-onset sporadic AD is extracellular deposits of amyloid beta (Aβ) peptides [3–5]. Aβ accumulations, as well as other insults in the brain, are a natural part of the aging process [6]. In AD, increased Aβ deposits compromise the immune function and clearance in a cyclic manner. Monomeric Aβ can self-aggregate to form oligomers, protofibrils, and amyloid fibrils which deposit as amyloid plaques. One hypothesis is that increased levels of Aβ leads to the hyperphosphorylation of the microtubule-associated protein tau, which results in formation of neurofibrillary tangles and accumulation of tau in some forms of AD [7, 8]. In addition to forming plaques and tangles, Aβ and tau can be recognized by pattern recognition receptors found on astrocytes and microglia/macrophages, and trigger an immune response characterized by release of inflammatory mediators which can increase Aβ production in a cyclic manner [9]. Furthermore, the increased inflammatory response also triggers peripheral inflammation. A significant body of evidence supports the hypothesis that increased peripheral inflammation can lead to increased neurodegeneration and may be a key driver of accelerated disease progression in AD [10]. Recent evidence from genomic studies has highlighted the role of the immune system in the etiology of AD [11–13]. Both the innate and adaptive immune responses have been implicated.

Though advancing age is the major risk factor for AD, it is not clear why certain aged individuals are more efficient at clearing Aβ and preventing neuroinflammation. Differences in the immune responses to Aβ between AD patients and healthy controls may be one of the factors. However, age-associated changes in the immune response to Aβ, which is a self-antigen, remains a relatively unexplored area of research. This is especially important since advancing age significantly impacts innate and adaptive responses which alter the response to both self and foreign antigens. Previous studies from our laboratory have demonstrated that the functions of important immune cells, dendritic cells (DCs), are substantially altered with age and play a role in enhancing peripheral inflammation [14–17]. DCs play a major role in innate immunity and have important functions in the phagocytosis of pathogens and cell debris [18]. DCs sense and respond to pathogens and endogenous danger signals by upregulating the antigen-presenting markers MHCII and costimulatory markers, as well as by secreting proinflammatory cytokines and priming T-cell responses. DC numbers have been reported to be increased in the brains of aged mice [19] while the percentage of DCs in the periphery is reported to be comparable between aged and young mice and humans [14, 20]. However, DCs from aged subjects display enhanced proinflammatory cytokines, such as tumor necrosis factor (TNF)-α and interleukin (IL)-6 secretion in response to Toll-like receptor (TLR)4 ligand and lipopolysaccharide (LPS), while the secretion of the anti-inflammatory cytokine IL-10 is impaired [14, 16]. Furthermore, we have also observed enhanced DC responses against self-antigens in the elderly [15]. The effect of age on T-cell responses has been studied more extensively. A decline in naive T cell numbers and regenerative capacity as well as a decrease in the diversity of the T-cell repertoire are a hallmark of aging [21, 22]. The numbers of naive and memory CD4^+^ T cell numbers are intact but there is an accumulation of CD4^+^CD28^−^ cells [21]. Furthermore, aging also impairs B cell number and function. Although the overall antibody production is reduced, there is increased production of low-affinity antibodies due to decreased isotype switching [23]. Altogether, these factors have a significant impact on the immunity of the aged individuals which may play a role in the development and progression of AD.

A clearer understanding of the immune response changes in aged individuals without AD will be helpful in identifying mechanisms that lead to production or clearance of Aβ. We therefore investigated age-associated changes in the response of monocyte-derived DCs as well as the T-cell memory and B-cell antibody response to Aβ peptide 1–42 (Aβ42) from aged subjects. Aβ42 is the most amyloidogenic form of the peptide according to the amyloid cascade hypothesis.

## Methods

### Aged and young blood donors

Peripheral blood samples were obtained from healthy aged and young volunteers. The young donors were aged between 20 and 35 years. Healthy aged donors were aged between 60 and 90 years. Subjects were recruited by the Institute of Clinical and Translational Science (ICTS), UC Irvine. Blood was drawn by research-trained nurses. The elderly subjects have a middle-class socioeconomic status and are living independently. Subjects suffering from diseases such as diabetes and heart disease or those on long-term medications as well as those taking drugs that can affect the immune system were excluded from the study. The number of subjects used for the different experiments differs since serum samples were available for more subjects than those used for cell-based experiments. The number of samples used for each experiment is specified in the figure legends. The inclusion and exclusion criteria of subjects for both types of experiment were the same. A description of the cohort is provided in Table 1 and includes samples for both serum and cellular experiments. This study was approved by the Institutional Review Board of the University of California, Irvine (UCI), and subjects provided their consent for the study.

**Table 1:** Description of aged and young cohorts

	Young	Aged
Number of subjects (*n*)	50	50
Age range (years)	20–37	66–95
Mean age (years)	26	78
Gender (*n*)		
Male	23	12
Female	27	38
Comorbidities (*n*)		
Osteoarthritis		28
Hypertension		14
Dyslipidemia		6
Medications (*n*)		
Vitamins and antioxidants		48

### Serum samples from patients and controls

De-identified serum samples from AD patients and patients with mild cognitive impairment (MCI), as well as age- and sex-matched healthy controls (HC), were obtained from the Alzheimer’s Disease Research Center (ADRC) core at UCI. The ADRC at UC Irvine is one of the 30 centers funded by the National Institutes of aging (NIA). The physicians in the Clinical Core of ADRC evaluate people with and without cognitive problems using the Clinical Dementia Rating (CDR) scale. Subjects are evaluated at least annually using neurological and physical examination and neuropsychological assessment. Brain imaging (positron emission tomography (PET) and magnetic resonance imaging (MRI)) is also performed to detect lesions. Amyloid levels in the cerebrospinal fluid (CSF) as well as blood and diagnostic tests are performed for diagnosis. In addition, an interview with a study partner is also conducted. The subjects are followed at least annually until death to document the normal and pathological brain changes as well as progression of the disease. Table 2 provides the description of the samples.

**Table 2:** Description of the Alzheimer’s disease (AD), mild cognitive impairment (MCI), and healthy control (HC) cohorts

	AD	MCI	HC
Number of subjects (*n*)	26	26	26
Age range (years)	75–87	74–86	75–85
Mean age (years)	79.6	79.6	79.3
Gender (*n*)			
Male	13	13	13
Female	13	13	13
MMSE score (range)	6–25	21–30	27–30
MMSE score (mean ± SD)	17.93 ± 5.5	27.5 ± 2.4	29.6 ± 0.8
CDR (range)	4.5–13	0.5–4.5	0–0.5
CDR (mean ± SD)	8 ± 3.1	1.97 ± 1.3	0.07 ± 2.1

*CDR* Clinical Dementia Rating, *MMSE* Mini-Mental State Examination, *SD* standard deviation

### DC generation and activation

Monocyte-derived DCs were prepared as previously described [15]. Briefly, purified monocytes from the aged and young individuals were cultured with granulocyte/macrophage colony-stimulating factor (GM-CSF) and IL-4 (PeproTech, NJ) for 6 days as previously described [15, 24]. Differentiated DCs were characterized as CD14^−^, CD11c^+^, and HLA-DR^+^ using flow cytometry after 6 days, indicating successful differentiation to DCs. Immature DCs from aged and young subjects were subsequently stimulated with Aβ42 (Tocris), Aβ fibrils (a kind gift from Dr. Blurton-Jones), recombinant human Tau protein (1 to 441) (Abcam; ab199583), nonaggregated isoform-F in serum-free AIM-V medium (ThermoFisher). Aβ fibrils were generated and characterized as previously described [25]. Briefly, the Aβ peptide was first dissolved in NH_4_OH (0.1%) to 1 mg/ml and then further diluted to 100 μg/ml using sterile endotoxin-free water, vortexed thoroughly, and incubated at 37 °C for 7 days. Aβ fibril conformation was verified via dot-blot and using conformation-specific antibodies as previously described [25]. The Aβ fibrils were mixed thoroughly before addition to cells. We cannot rule out that the preparation may contain some oligomers. Optimal activation of DCs was observed at 10 μg/ml concentration of these peptides. After overnight stimulation, supernatants were collected and assayed for IL-6, IL-1β, C-X-C motif chemokine 10 (CXCL-10), C-C motif chemokine ligand (CCL)-2 (BD Biosciences, San Jose, CA), and CCL-4 (R&D Systems) using specific enzyme-linked immunosorbent assays (ELISAs). A disintegrin and metalloproteinase domain (ADAM)12 was assayed by an ELISA kit from R&D Systems while brain-derived neurotrophic factor (BDNF) was assessed by a specific ELISA from Biosensis.

### DC and T cell coculture

Stimulated DCs (2 × 10^4^) were cultured with purified, naive, allogeneic T cells (1 × 10^5^) from young donors at a ratio of 1:5 for 6 days. Naive CD4 T cells were isolated by negative selection using a magnetic bead-based kit (Stemcell Technologies). The purity of the naive T-cell (CD4^+^, CD45RA^+^, CCR7^+^) preparation was confirmed with flow cytometry. DC/T-cell supernatant was collected and assayed for interferon (IFN)-γ, TNF-α, IL-10 (BD Biosciences), and IL-17 (R&D Systems) using specific ELISAs.

### PBMC stimulation

Peripheral blood mononuclear cells (PBMCs) from aged and young subjects were stimulated with Aβ42 in serum-free AIM-V medium for 6 days. Supernatants were collected and assayed for IFN-γ, TNF-α, IL-10, and IL-17 using specific ELISAs.

### Aβ antibody assay

Previously collected plasma samples from aged and young subjects and serum samples from AD, MCI, and age-matched controls were assayed for the presence of Aβ42-specific antibodies using an in-house ELISA which was based on a previously published assay by Qu et al. [26]. Briefly, ELISA (Maxisorp) plates were coated with 2 μg/ml human Aβ42 peptide in a 0.1 M bicarbonate-carbonate buffer (pH 9.0) at 4 °C overnight (100 μl/well). The wells were then blocked with 100 μl 1% bovine serum albumin (BSA) in phosphate-buffered saline (PBS) for 1 h and then washed three times with PBST (PBS containing 0.05% (v/v) Tween-20). Then, 100 μl of plasma (diluted 1:5 with blocking buffer) was added to the plates and incubated at room temperature for 2 h. The plates were then washed three times with PBST and incubated for 2 h at room temperature with the secondary antibody conjugated to streptavidin (eBioscience) at a 1:1000 dilution in 1% BSA-PBST. The plates were then washed five times with PBST and incubated with streptavidin HRPO (eBioscience, 1:1000) for 30 mins. After washing, a 100-μl solution of 3,3,5,5-tetramethylbenzidine (TMB) was added to the wells to form a colored reaction product indicating the presence of anti-Aβ antibodies. The reaction was stopped by adding 2 N sulfuric acid and absorbance was measured at a wavelength of 450 nm with a plate reader. To determine the specificity of binding of antibodies to the Aβ peptide, a scrambled peptide of Aβ (a randomly scrambled amino-acid sequence of Aβ42 peptide) was used as a control. The binding to the scrambled peptide was a measure of nonspecific binding. The values were calculated as a ratio of absorbance to Aβ peptide and scrambled peptide. Ratios above one were considered positive.

### Statistical analysis

Statistical analysis was performed using GraphPad Prisms software. Data were checked for normality, and significance within groups was measured by *t* tests. The unpaired *t* test was used to measure significance between aged and young subjects. Values of *p* < 0.05 were considered significant. For comparison between three or more groups, one-way analysis of variance (ANOVA) followed by Tukey’s test was used. All tests were two tailed with 95% confidence intervals.

## Results

### DCs from aged subjects are weakly reactive to Aβ peptide

**Fig. 1:**
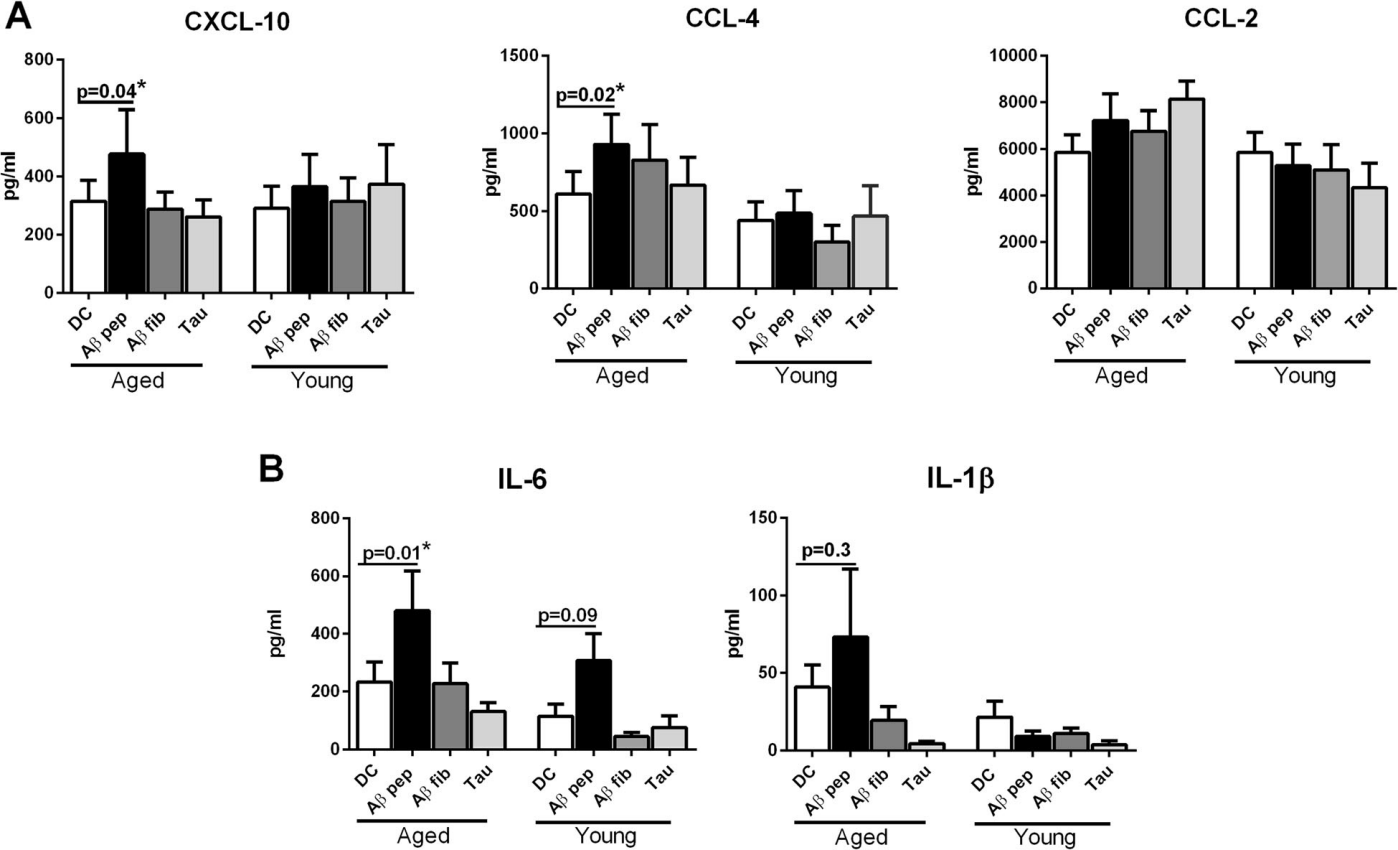
Dendritic cells (DCs) from aged subjects display increased secretion of inflammatory chemokines and cytokines in response to amyloid-beta (Aβ) peptide. DCs from healthy aged and young subjects were cultured overnight with Aβ42 peptide (pep), Aβ fibril (fib), and tau protein at 10 μg/ml. Cytokine secretion in the supernatants was quantified by ELISA. Bar graphs depict the levels of **a** chemokines (C-X-C motif chemokine 10 (CXCL-10), C-C motif chemokine ligand (CCL)-4, and CCL-2) and **b** cytokines (interleukin (IL)-6 and IL-1β). Results are shown as mean ± SEM of 18 different aged and 18 different young subjects. *p* values are based on two-tailed, parametric paired *t* tests with 95% confidence interval. Comparison of stimulated DCs in each group was performed against the control of the same age group

Aging significantly impacts DC phenotype and functions such as antigen presentation and chemokine/cytokine secretion. We compared the response of DCs from healthy aged and young subjects to Aβ42 (Aβ peptide) to examine the role of DCs in AD disease progression. Supernatants of Aβ-stimulated DCs from an initial three experiments were screened with a multiplex kit detecting thirty different chemokines and cytokines. In addition, we also determined the levels of ADAM12 and BDNF as these are both secreted by DCs and are involved in AD [27, 28]. A response to Aβ was only observed for CXCL-10, CCL-4, CCL-2, IL-6, and IL-1β (Additional file 1: Figure S1). Subsequent experiments revealed that DCs from aged subjects exhibited significantly increased secretion of CXCL-10 (*p* = 0.04), CCL-4 (*p* = 0.02), and IL-6 (*p* = 0.01) in response to Aβ peptide relative to DCs from young subjects (Fig. 1). The levels of IL-1β and CCL-2 secreted were not significantly different between aged and young DCs.

In addition to Aβ peptide, we also compared the response of DCs from aged and young subjects to Aβ fibril and tau proteins. However, no significant difference in chemokine or cytokine levels was observed with Aβ fibril and tau (Fig. 1). Taken together, these results demonstrate that DCs from healthy aged subjects are weakly responsive and generate a low-level mild chemokine response to Aβ peptide compared with DCs from young subjects.

### Aβ peptide-activated DCs from aged subjects do not prime CD4 T cells to secrete cytokines

**Fig. 2:**
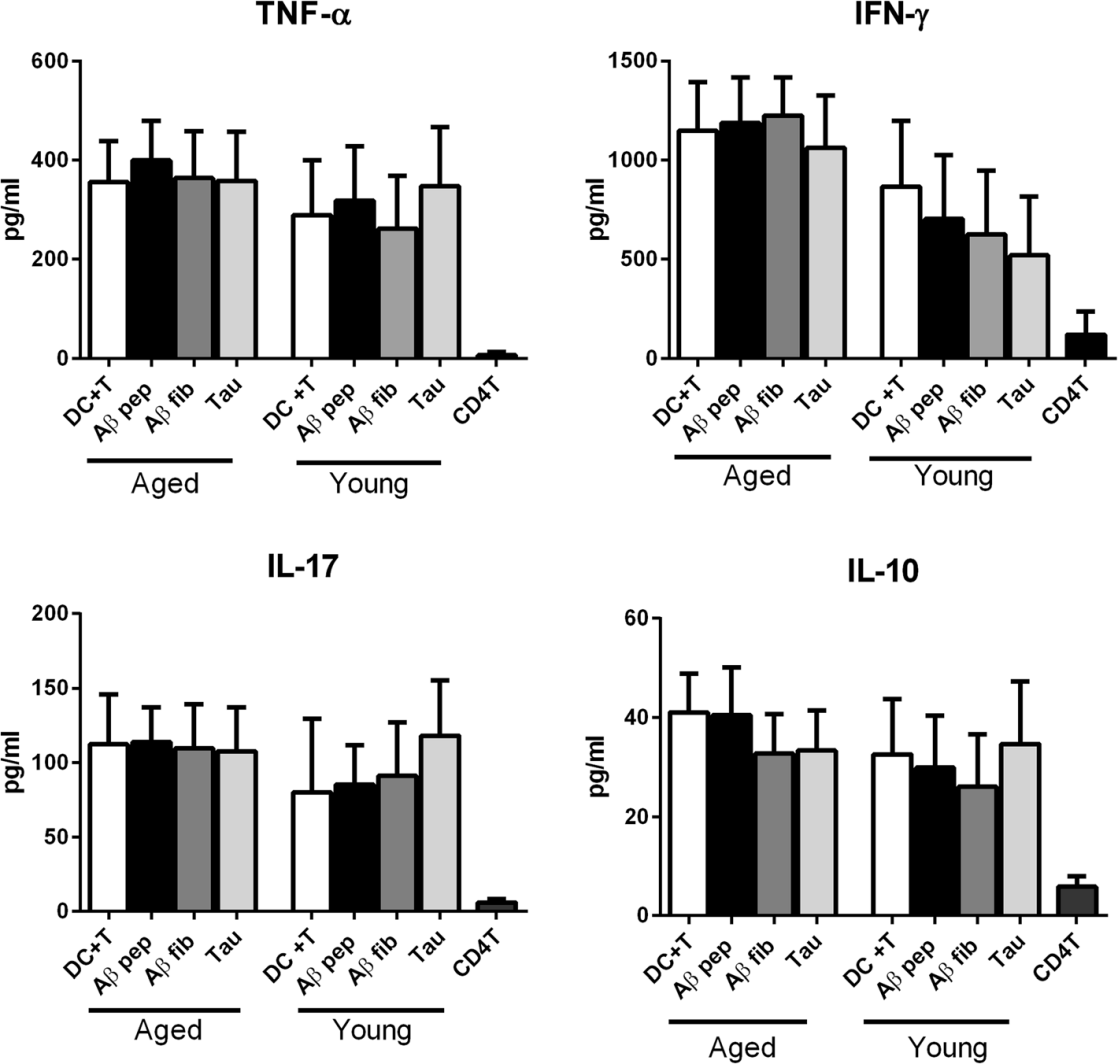
Amyloid-beta (Aβ) activates dendritic cells (DCs) from aged subjects which prime CD4 T cells to secrete tumor necrosis factor (TNF)-α. Aβ42 peptide (pep)-, Aβ fibril (fib)-, and tau protein-exposed DCs from aged and young subjects were cultured with purified CD4 T cells for 6 days. Bar graphs depict the level of the cytokines TNF-α, interferon (IFN)-γ, interleukin (IL)-17, and IL-10 in the supernatants after 6 days as determined by specific ELISA. Results are shown as mean ± SEM of 14 different aged and 14 different young subjects. *p* values are based on two-tailed, parametric paired *t* tests with 95% confidence interval. Comparison of stimulated DCs in each group was performed against the control of the same age group

Cytokines and chemokines secreted by DCs dictate the polarization of T-helper cell responses towards Th1/Th2/Treg/Th17 T-cell subtypes and their secretion of inflammatory cytokines. Therefore, we determined the effect of Aβ peptide on CD4 T-helper cell differentiation by aged and young DCs. Purified, naive CD4 T cells from young subjects (to exclude any T-cell defect) were cultured along with aged and young DCs stimulated with the aforementioned peptide. Cytokine secretion by T cells was quantified from culture media using ELISA. Secretion of TNF-α, IFN-γ, IL-17, and IL-10 by T cells was not significantly different between stimulated and unstimulated DCs in both age groups (Fig. 2). In summary, these data indicate that Aβ peptide-, Aβ fibril-, and tau-activated DCs from aged subjects do not induce significant priming of CD4 T cells. This is in keeping with the poor response of DCs from aged subjects to Aβ peptide observed in Fig. 1.

### Aβ peptide does not induce T cell cytokine secretion from PBMCs of aged and young subjects

**Fig. 3:**
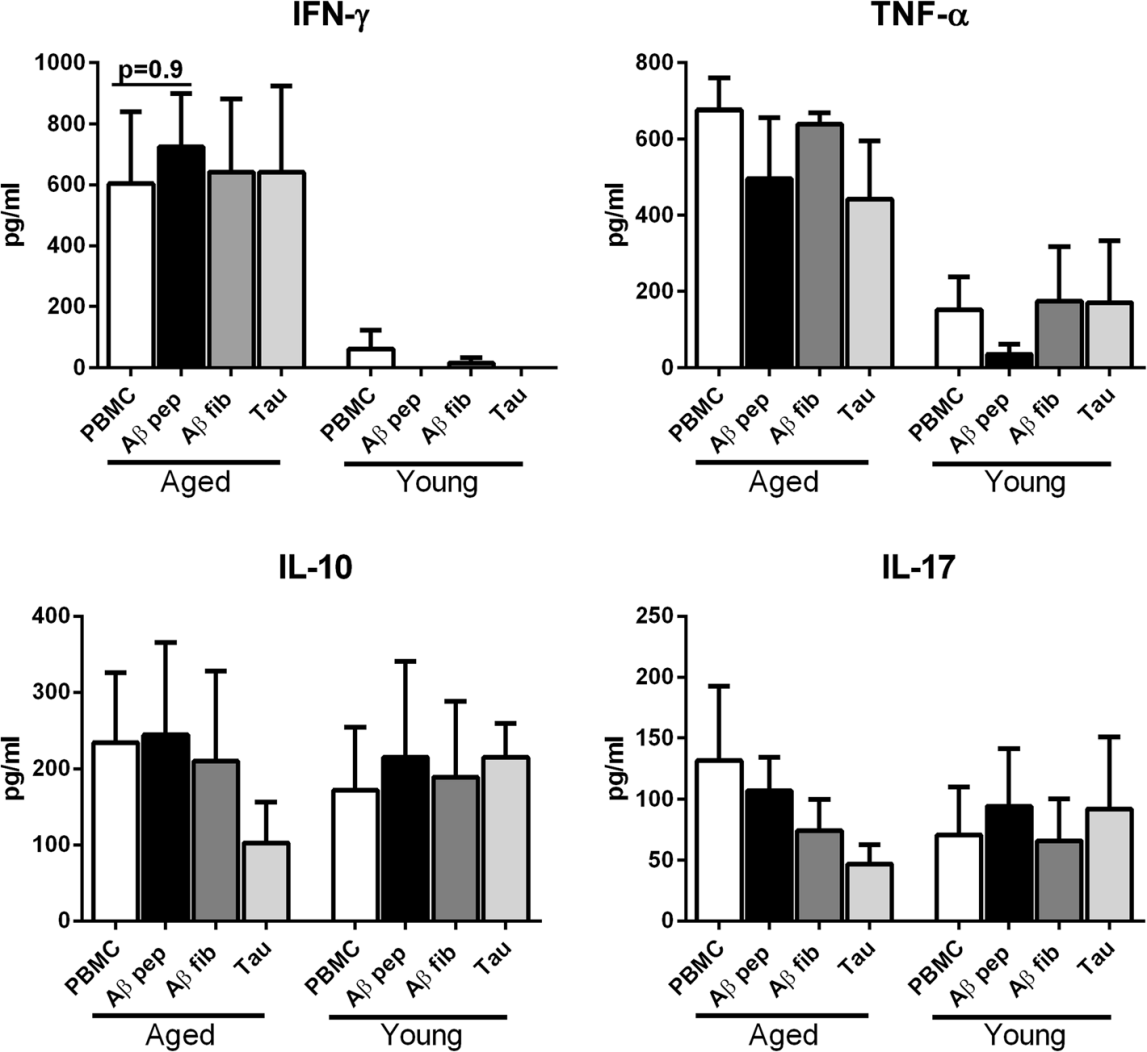
Amyloid-beta (Aβ) peptide does not induce T cell cytokine secretion from peripheral blood mononuclear cells (PBMCs) of aged and young subjects. PBMCs from aged and young subjects were stimulated with Aβ42 peptide (pep) for 6 days. Bar graphs depict the level of the cytokines tumor necrosis factor (TNF)-α, interferon (IFN)-γ, interleukin (IL)-17, and IL-10 in the supernatants after 6 days as determined by specific ELISA. Results are shown as mean ± SEM of 6 different aged and 6 different young subjects. *p* values are based on two-tailed, parametric paired *t* tests with 95% confidence interval. Comparison of stimulated DCs in each group was performed against the control of the same age group. fib fibril

To confirm that the poor CD4 T-cell priming observed above is not an artifact of in-vitro coculture, we determined whether Aβ-specific memory T cells were present in aged subjects. The generation and persistence of memory T cells would indicate a pre-existing efficient immune response to Aβ strong enough to generate T-cell memory for subsequent encounters. To investigate this, PBMCs from aged and young subjects were stimulated with Aβ peptide for 6 days to reactivate any potential memory T cells. Supernatants collected were assayed for TNF-α, IFN-γ, IL-10, and IL-17 as a measure of T-cell activation. Stimulation with Aβ did not induce secretion of the cytokines above those with the unstimulated PBMCs in either aged or young subjects suggesting that the numbers of memory T cells to Aβ are either very low or absent in both populations (Fig. 3). We did observe significantly increased (*p* < 0.05) secretion of IFN-γ and TNF-α from unstimulated PBMCs of aged subjects compared with young subjects, which is in line with the enhanced inflammatory state of aged subjects as previously reported [24].

### Aβ42 peptide-specific antibodies of IgM isotype are increased in aged subjects

**Fig. 4:**
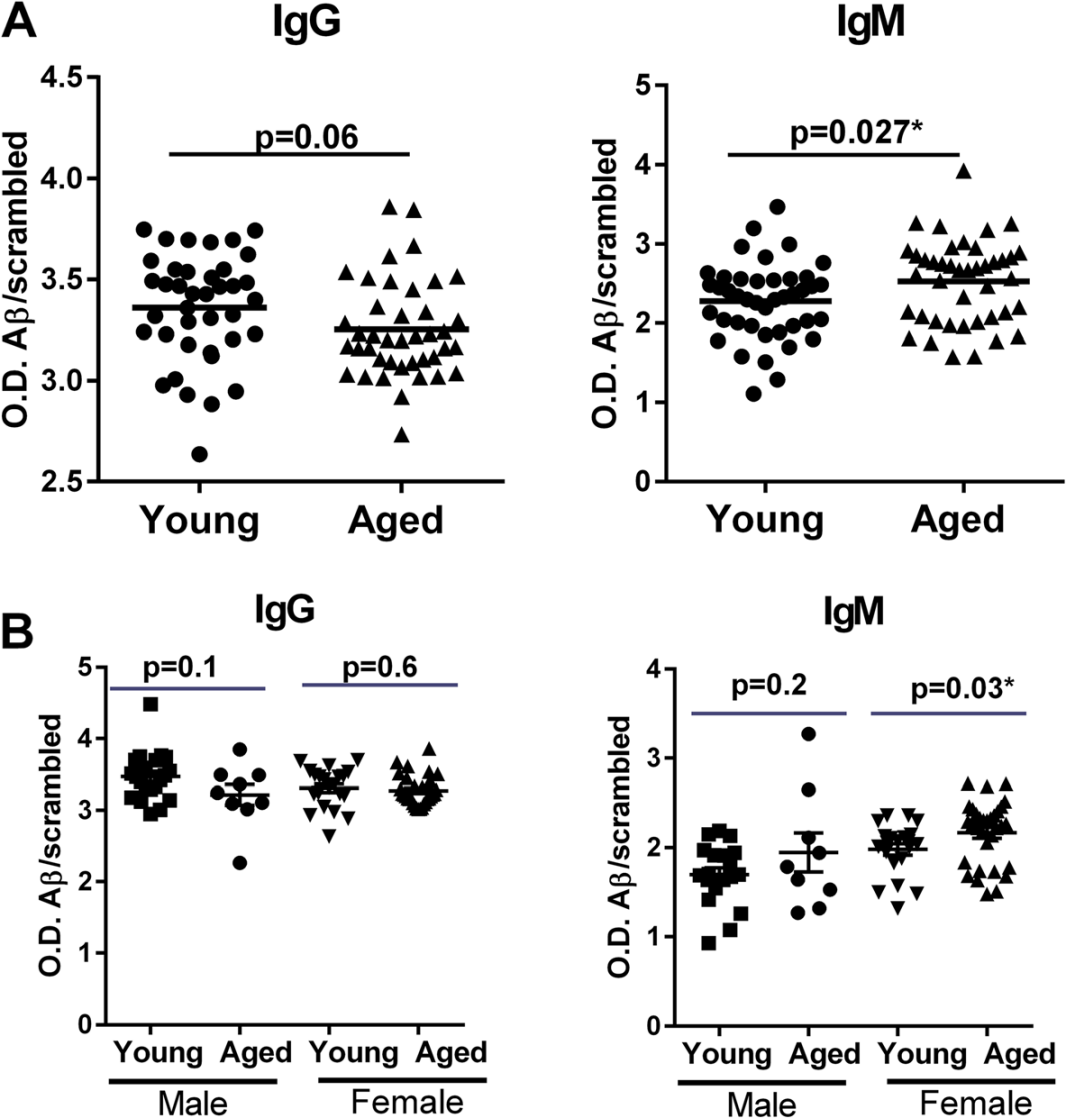
Amyloid-beta (Aβ)42 peptide-specific antibodies of IgM isotype are increased in aged subjects. The levels of Aβ42-specific antibodies as well as Aβ scrambled peptide were measured from the plasma samples of aged and young subjects using an in-house ELISA. **a** Dot plots depict the ratio of Aβ-specific IgG and IgM antibodies to scrambled peptide antibodies. Results are shown as mean ± SD of 43 different aged and 43 different young subjects. **b** Dot plots depict the ratio of Aβ42-specific IgG and IgM antibodies to scrambled peptide antibodies in male and female aged and young subjects. Results are shown as mean ± SD of 9 aged males and 20 young males and 34 aged females and 23 young females. *p* values are based on two-tailed, unpaired *t* tests with 95% confidence interval. O.D. optical density

Next, we determined whether there is a change in Aβ antibody levels with age. We assayed the levels of both IgG and IgM isotypes of antibodies against Aβ42 in the plasma from males and females of aged and young subjects using an in-house ELISA. The IgM class was assayed because we did not observe a T-cell memory response in PBMCs of aged and young subjects; thus, it was possible the T-cell-independent IgM antibodies may be affected by age. An increase in natural autoantibodies of the IgM class has been reported with age [29, 30]. The presence of Aβ-specific antibodies were assayed using an in-house ELISA. As shown in Fig. 4a, we observed a tendency towards a decrease in Aβ-specific IgG antibodies (*p* = 0.06) in the aged subjects compared with the young subjects. In contrast, there was a significant increase in Aβ-specific IgM antibodies in the aged subjects (*p* = 0.02). Gender-based analysis revealed that the Aβ-specific IgG antibody levels were similar in both males and females of aged and young subjects. However, IgM levels were significantly higher (*p* = 0.02) in aged females compared with young females while no significant difference was observed in males (Fig. 4b). Altogether, these results indicate that low-affinity antibodies of IgM against Aβ are increased with age, particularly in females.

### Aβ42 peptide-specific antibodies of IgM isotype are decreased in AD patients

**Fig. 5:**
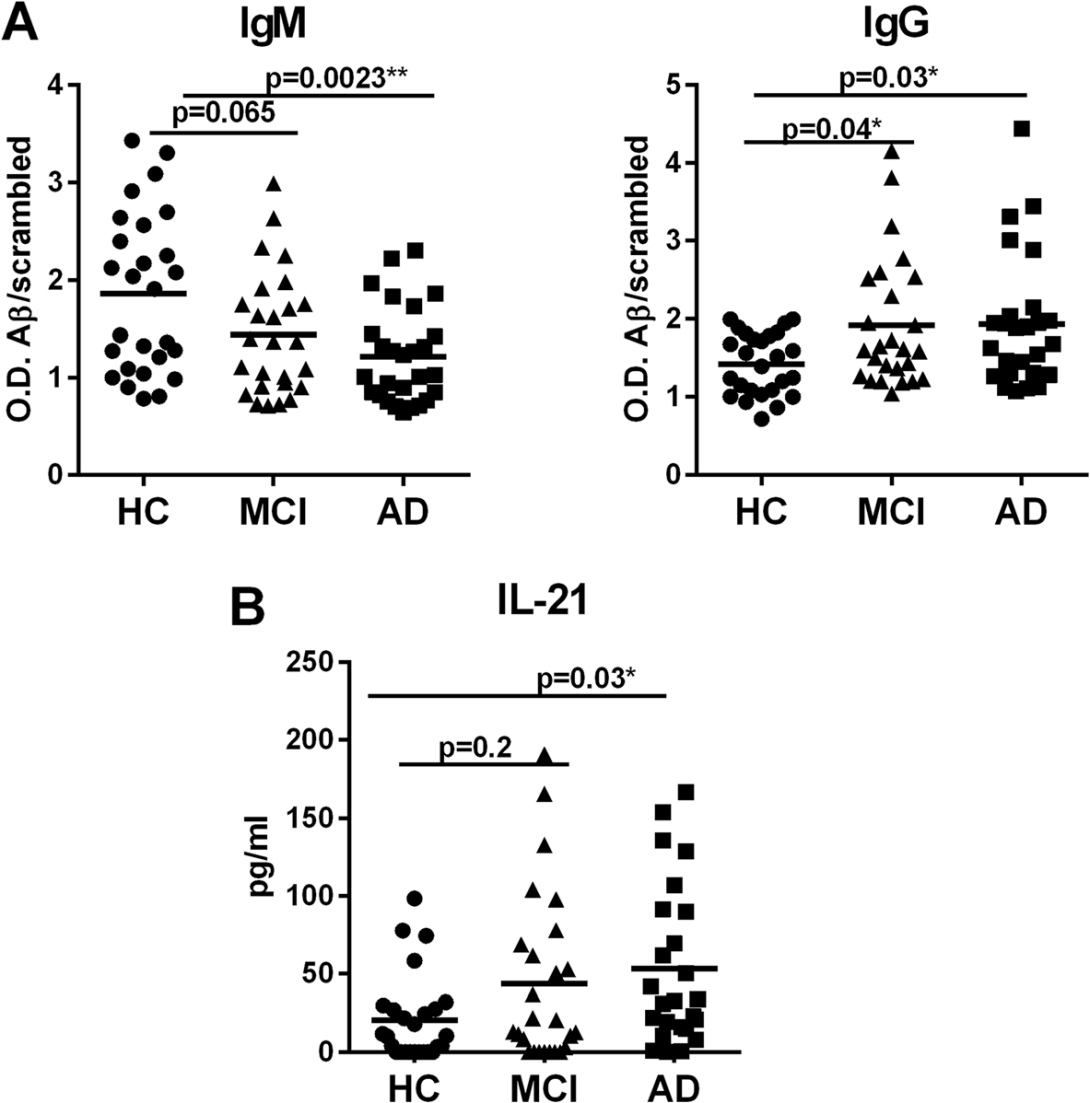
Amyloid-beta (Aβ)42 peptide-specific antibodies of IgM isotype are decreased in Alzheimer’s disease (AD) patients compared with healthy controls (HC). The levels of Aβ42-specific antibodies as well as Aβ scrambled peptide were measured from the plasma samples of AD patients, those with mild cognitive impairment (MCI), and age-matched healthy controls. **a** Dot plots depict the ratio of Aβ42-specific IgG and IgM antibodies to scrambled peptide antibodies. **b** Dot plot depicts the levels of IL-21 in the same samples. Results are shown as mean ± SD of 26 AD patients, 26 MCI patients, and 26 healthy controls. *p* values are based on one0way ANOVA followed by Tukey’s test with two tails and 95% confidence interval

If the increased levels of Aβ-specific IgM antibodies observed in aged subjects are assumed to be protective, then the levels of these antibodies should be decreased in AD patients. To confirm this, we determined the levels of Aβ42-specific IgM and IgG antibodies in the serum samples from AD and MCI patients as well as age- and sex-matched healthy controls obtained from the ADRC core at UCI. As shown in Fig. 5, the level of IgM antibodies was significantly decreased in the serum of AD patients versus the healthy controls (*p* = 0.0023). MCI patients also displayed a tendency towards decreased levels of IgM compared with healthy controls, but the difference was not significant (*p* = 0.065). The level of IgM antibodies between MCI and AD patients was also not significant. In contrast to IgM, the IgG antibodies displayed a significant increase in both MCI (*p* = 0.04) and AD patients (*p* = 0.03) compared with the controls.

T follicular helper (Tfh) cells play a major role in class switching of antibodies from IgM to IgG and other subclasses [31]. The signature cytokine for Tfh cells is IL-21 which acts on B lymphocytes to induce class switching and antibody secretion. Therefore, the level of IL-21 in the serum of control, MCI, and AD patients was assayed using a specific ELISA. Remarkably, we observed a significant increase in the IL-21 levels in the serum of AD patients compared with controls (*p* = 0.03). IL-21 also displayed an increasing trend in MCI patients compared with controls. Since AD prevalence is higher in women [32], data were also analyzed to investigate the effect of gender differences on the levels of IgG and IgM antibodies. No significant differences were observed (data not shown). Thus, both the IgG subclass and IL-21 are increased in AD patients compared with controls.

## Discussion

In this, we examined the immune response of the healthy elderly to Aβ. Our results indicate that DCs from healthy aged subjects primarily produce chemokines in response to Aβ peptide compared with DCs from young subjects (Fig. 1). One of the reasons for the increased response of DCs from aged subjects to Aβ could be the increased basal level of activation of DCs which increases their reactivity to self-proteins, as observed in our earlier studies [15]. The increased levels of CCL-4 and CXCL-10 produced by DCs from aged subjects can attract a variety of immune cells including monocytes and natural killer (NK) cells which can help with the clearance of Aβ. Furthermore, DCs from healthy aged subjects display only low-level inflammatory responses to Aβ which is beneficial since mild activation of microglia and astrocytes has been reported to have neuroprotective effects and ameliorates early symptoms of neurodegeneration [33]. Previous studies have compared the response of DCs from AD subjects with healthy aged controls to Aβ and observed an increased inflammatory response in the DCs from AD subjects [27]. They also observed a decrease in the secretion of BDNF by DCs from AD subjects compared with controls. We did not observe induction of BDNF by Aβ in our studies (Additional file 1: Figure S1) in either age groups. One reason for this discrepancy could be due to difference in stimulatory conditions since, in the previous study, the DCs were stimulated in the presence of GM-CSF and IL-4 (factors used for DC differentiation). In contrast to Aβ peptide, Aβ fibrils and tau protein did not activate DCs. The reason for this is not clear, but it is possible that a much higher concentration of these may be required to activate DCs. It may be that the enhanced inflammatory response as reported for AD subjects is detrimental since high levels of inflammation have been reported to promote the production of Aβ [9].

In keeping with the weak response to Aβ, DCs from aged subjects also did not induce cytokine secretion from T cells (Fig. 2). In contrast, a previous study by Ciaramella et al. [34, 35] reported that differentiation of monocytes from healthy subjects to DCs in the presence of Aβ42 resulted in a decrease in MHC expression and the ability to activate T cells. The decrease in T-cell priming by the DCs in this earlier study could be due to different conditions of exposure to Aβ (during differentiation versus already differentiated DCs). DCs isolated from AD patients and subsequently stimulated with LPS have also been demonstrated to have reduced antigen-presenting ability [34] compared with controls. Reduced priming of T cells by DCs from AD patients may enhance the induction of T regulatory cells (Tregs), which could explain the increased levels of Tregs observed in the periphery of MCI and AD patients [36]. Induction of Tregs in the periphery has been shown to be detrimental for AD since transient depletion of Tregs in the circulation ameliorated the brain pathology and reversed cognitive decline in mouse models of AD [37]. Furthermore, myeloid DC numbers are also reported to be decreased in AD patients [38]; low myeloid DC levels could further decrease the immune response against Aβ in the periphery and prevent its clearance. In light of these observations, we can hypothesize that the low-level reactivity of DCs from healthy aged subjects to Aβ aids in its clearance and prevents inflammation. However, further studies with DCs from AD patients at different stages of the disease need to be performed to draw any conclusions. Also, DCs in the periphery do not represent the brain, and thus the data may represent primarily systemic effects. Further studies using mouse models may be able to correlate brain inflammation with systemic effects. One of the other limitations of the study is the use of high concentrations (10 μg/ml) of Aβ42. This is higher than the pico/nano levels observed in the circulation [39]. That being said, the total concentration of all Aβ 38–42 peptides may be much higher. Also, the levels in the brain are much higher. We have only examined the reactivity to Aβ42 peptide; Aβ40 is also a highly pathogenic form along with Aβ43, 38, and so forth. It would be interesting to compare the immune response against these different forms although, since the peptide sequences are similar, the different Aβ peptides are expected to induce comparable DC responses. Similar response of DCs to hyperphosphorylated tau and tangles may also be different and differ as the disease progresses. Another thing to keep in mind is that this study is a case-control study, and it is not known how these findings will translate to populations where relationships between dementia status and neuropathological change are more complex.

In this study, we also observed an increase in Aβ-specific IgM antibodies in the circulation of healthy elderly (Fig. 5) compared with AD patients, while the level of Aβ-specific IgG antibodies was decreased. The presence of high levels of IgM antibody along with low levels of IgG antibodies also indicates a weak immune response. It suggests that, although the immune response against Aβ is initiated in the healthy controls, it is not strong enough to induce class switching to IgG or the generation of T cell memory [40–42]. Increased levels of IL-21 found in the serum of AD patients also supports this since IL-21 enhances the differentiation of B cells towards antibody-secreting B cells and also helps in class switching of IgM antibodies towards IgG antibodies. Antibodies against self-proteins such as Aβ are called natural antibodies. Natural antibodies are essentially antibodies of the IgM isotype present in the circulation of normal humans and other mammalian species. They are detectable in the serum of healthy individuals before deliberate immunization. They have been found to play an innate-like role in protection against infectious agents and to exert homeostatic functions in a variety of experimental models [43–45]. One of the major homeostatic functions of the natural antibodies is to help in the removal of autoantigens such as Aβ. The IgM isotype is beneficial in this regard as these antibodies have a low affinity against the antigen compared with IgG antibodies. However, their avidity of binding to antigens is high because of their pentameric structure which aids in clearance of the antigens. Natural autoantibodies against Aβ have also been demonstrated to play a protective role in AD. Autoantibodies against Aβ play a role in Aβ clearance and these are lower in AD patients than controls [46, 47]. Very recently, a study by Marsh et al. [46] demonstrated that genetically modified AD mice lacking three key immune cell types (T cells, B cells, and NK cells) displayed a twofold increase in Aβ accumulation compared with AD mice with an intact immune system [46]. They further showed that this is due to a decrease in antibodies as the accumulation of natural antibodies in the brain in immune competent mice helped increase the clearance of Aβ. Antibody production thus appears to be beneficial in the fight against AD.

The increased levels of IL-21 observed in AD patients is also indicative of ongoing inflammation since, in addition to B-cell differentiation, IL-21 is also a highly inflammatory cytokine that can enhance the differentiation of IL-17, producing Th17 cells [48]. Both IL-21 and IL-17 have been implicated in numerous inflammatory diseases [30, 31, 48, 49]. For example, high levels of IL-21 have been demonstrated to promote a range of autoimmune diseases, including multiple sclerosis, inflammatory bowel disease, and psoriasis. More recently, IL-21 was found to be highly upregulated in the mouse brain after cerebral ischemia [50]. It is therefore plausible to speculate that IL-21 may play a dual role in the pathogenesis of AD by enhancing IgG production and by increasing inflammation via Th17 cells. Enhanced production of Aβ and related peptides, fibrils, and tangles in AD may also act as antigens to induce inflammatory immune responses in addition to causing neurological damage as suggested by the amyloid cascade hypothesis. The presence of T cells in the brain of AD patients [46] may be indicative of disruption of the blood-brain barrier due to increased peripheral inflammation. It would be interesting to determine a correlation between IL-21 levels as well as IL-21-producing Tfh cells with disease progression.

## Conclusions

In summary, we demonstrate for the first time that DCs from healthy aged subjects display mild inflammatory responses to Aβ in the form of chemokine secretion and low-level T-cell priming which may facilitate clearance of Aβ. Furthermore, we also observe enhanced levels of Aβ-specific IgM antibodies in the circulation of healthy controls compared with AD subjects, which may also help clear the Aβ. Thus, we describe two novel mechanisms which may be operating in healthy subjects to prevent the development of AD. These data also suggest novel therapeutic strategies using antibodies to clear Aβ. In addition, we also observe increased IL-21 in the serum of AD patients, which may serve as a biomarker for immune and inflammatory response in these subjects.

## Additional file

Additional file 1: Factors secreted by DCs after stimulation with Aβ. (JPG 63 kb)
