# Supplementary figures and images for: IgM response against amyloid-beta in aging: a potential peripheral protective mechanism
Source: Alzheimers Res Ther. 2018 Aug 16;10:81. doi: 10.1186/s13195-018-0412-9 (PMC6097437; doi:10.1186/s13195-018-0412-9)

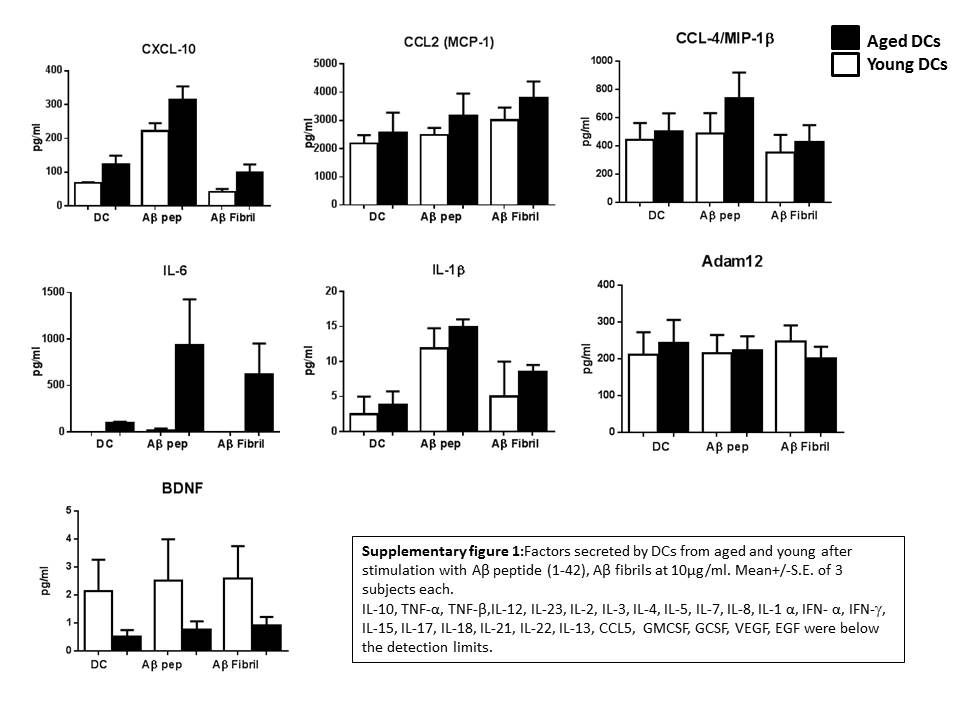

Supplement: Supplementary file 1 — Factors secreted by DCs after stimulation with Aβ. (JPG 63 kb) [file 13195_2018_412_MOESM1_ESM.jpg]
